# Depressive rumination and the emotional control circuit: An EEG localization and effective connectivity study

**DOI:** 10.3758/s13415-016-0456-x

**Published:** 2016-08-29

**Authors:** Magdalena A. Ferdek, Clementina M. van Rijn, Miroslaw Wyczesany

**Affiliations:** 1 Donders Institute for Brain, Cognition, and Behaviour, Radboud University Nijmegen, Nijmegen, The Netherlands; 2 Psychophysiology Laboratory, Institute of Psychology, Jagiellonian University, PL-30060 Ingardena 6, Krakow, Poland

**Keywords:** Depressive ruminations, Emotional control, DIPFIT, Independent component clustering, DTF

## Abstract

Ruminations are repetitive thoughts associated with symptoms, causes, and consequences of one’s negative feelings. The objective of this study was to explore the neuronal basis of depressive rumination in a non-clinical population within the context of emotional control. Participants scoring high or low on the tendency to ruminate scale took part in the EEG experiment. Their EEG data were collected during a state of induced depressive ruminations and compared with positive and neutral conditions. We hypothesized that both groups would differ according to the level of activation and effective connectivity among the structures involved in the emotional control circuit. Clustering of independent components, together with effective connectivity (Directed Transfer Function), was performed using the EEG signal. The main findings involved decreased activation of the left dorsolateral prefrontal cortex (DLPFC) and increased activation of the left temporal lobe structures in the highly ruminating group. The latter result was most pronounced during the ruminative condition. Decreased information from the left DLPFC to the left temporal lobe structures was also found, leading to the conclusion that hypoactivation of the left DLPFC and its inability to modulate the activation of the left temporal lobe structures is crucial for the ruminative tendencies.

## Introduction

Self-reflection can have both light and dark sides. The ability to analyze one’s own mental states is unique to humans and seems to be highly adaptive for functioning in a complex world. Nonetheless, there is a form of self-reflection that has harmful consequences and can lead to the magnification and prolongation of depressive moods. Ruminative response style, according to Nolen-Hoeksema, Wisco, and Lyubomirsky (2008), is a form of responding to distress, which *“involves repetitively and passively focusing on symptoms of distress and on the possible causes and consequences of these symptoms.”* It is suggested that ineffective cognitive control over emotional information, accompanied by increased emotional reactivity to negative self-referential stimuli, underlie depressive ruminations (Kühn, Vanderhasselt, De Raedt, & Gallinat, 2012; Mandell, Siegle, Shutt, Feldmiller, & Thase, 2014). Indeed, ruminators are characterized by increased self-focus (Nejad, Fossati, & Lemogne, 2013) and memory/attentional biases towards negative stimuli. Highly ruminating individuals experience difficulties with diverting attention away from the negative material (Joormann & D’Avanzato, 2010) and recall more negative autobiographical memories than positive ones (Lyubomirsky, Caldwell, & Nolen-Hoeksema, 1998). As we assume that extensive rumination arises due to ineffective cognitive emotional control, in the present study we investigate the brain’s emotional control circuit and its relationships to ruminative tendencies in a non-clinical population.

Studying the tendency to ruminate is important in the context of susceptibility for developing depressive disorder, as it has been shown that ruminative style is a predictor of the onset of this condition (Nolan, Roberts, & Gotlib, 1998; Nolen-Hoeksema, 2000; Roberts, Gilboa, & Gotlib, 1998; Spasojević & Alloy, 2001). The maladaptiveness of a tendency to ruminate begins with the prolonged and unproductive analysis of one’s negative emotional state, which additionally intensifies the lowered mood. Finally, it creates a self-perpetuating, vicious cycle of a depressive state and ruminations. Furthermore, depressive ruminations do not give rise to active problem solving. People with a high tendency to ruminate remain fixated on negative emotions, usually without taking the necessary actions to solve the underlying problems. Women characterized by the ruminative response style procrastinate on seeing a specialist in spite of noticing evident symptoms of breast cancer (Lyubomirsky, Kasri, Chang, & Chung, 2006). The tendency to ruminate is defined quantitatively, mostly by the frequency of ruminations during the lowered mood state (Blaut & Paulewicz, 2011). As this tendency is relatively stable during the lifespan (Nolen-Hoeksema & Davis, 1999), it can be studied as an individual trait. In spite of its relative stability, it is still a sensible target in therapy, as its frequency can be lessened by psychological interventions. Taking these facts into consideration, it is crucial to study the tendency to ruminate as a factor that predisposes individuals to depressive disorder.

Emotional control relates to processes of creating a new or changing an ongoing emotional response (Ochsner & Gross, 2005). Despite the fact that many diverse strategies of emotional control exist, all of them seem to depend on a similar emotional control circuit, which comprises the regulatory loop between the prefrontal cortex, limbic cortex, and other limbic regions such as the amygdala and hippocampus (Ochsner, Bunge, Gross, & Gabrieli, 2002). Within this circuit, effective emotional control (down-regulation of negative emotional states) is manifested by higher activation of the prefrontal cortex and decreased activation of the limbic structures (Taylor & Liberzon, 2007), as well as increased connectivity of the prefrontal cortex, which possibly initiates the modulating actions (Wyczesany, Ferdek & Grzybowski, 2014; Wyczesany, Ligeza & Grzybowski, 2014). Supporting this assertion, it was found by Banks et al. (2007) that the fronto-limbic coupling may be a predictor of the successful top-down cognitive modulation of the negative affect. Unsuccessful top-down modulation of negative emotional state seems to be a key characteristic that underlies mood disorders such as depression (Joormann & Gotlib, 2008). Indeed, several functional magnetic resonance imaging (fMRI) studies revealed changes in the emotional control circuit in depressed patients. It was found that depressed participants had increased activity in the amygdala while they were trying to down-regulate their negative affect, which may indicate that the prefrontal cortex modulatory actions on the limbic regions were rather ineffective (Beauregard, Paquette, & Lévesque, 2006; Johnstone, van Reekum, Urry, Kalin, & Davidson, 2007). Depressed individuals are also characterized by decreased reactivity of the dorsolateral prefrontal cortex (DLPFC) in response to emotional stimuli (Hooley et al., 2009; Schaefer, Putnam, Benca, & Davidson, 2006), and they have abnormal resting-state functional connectivity within neural circuits that mediate emotional processing (Cullen et al., 2009). Based on all of this data, we conclude that impaired emotional regulation is the main factor triggering extensive depressive rumination. Thus, in the present study, we examine the characteristics of the fronto-limbic circuit in ruminating subjects and compare them with non-ruminating individuals. We predict that a crucial difference between ruminators and nonruminators lies in the effectiveness of the cognitive emotional control – the top-down modulation of the subcortical structures activation by the prefrontal cortex.

Neural correlates of depressive ruminations have been studied using neuroimaging methods. The prefrontal cortex, anterior cingulate cortex, and temporal lobe structures (amygdala, hippocampus) were shown to be related to depressive ruminations but the exact model of the tendency to ruminate requires further investigation (Cooney, Joormann, Eugène, Dennis, & Gotlib, 2010; Johnson, Nolen-Hoeksema, Mitchell, & Levin, 2009; Kühn et al., 2012; Mandell et al., 2014; Ray et al., 2005). Functional differences between ruminators and nonruminators in the activity of the DLPFC and the ACC regions are still unclear, with some of the studies showing an increase while others show a decrease in their activation. These discrepancies can be partly attributed to differences in conceptualizations of depressive ruminations across the research. Depressive ruminations were studied both as a phenomenon induced in laboratory conditions (Cooney et al., 2010) or as a personality trait (Kühn et al., 2012; Ray et al., 2005) in healthy individuals (Kühn et al., 2012) or in depressed patients (Cooney et al., 2010; Mandell et al., 2014; Ray et al., 2005). Ruminators’ and nonruminators’ brain measurements were recorded at rest (Kühn et al., 2012) or during a specific cognitive task (Johnson et al., 2009; Mandell et al., 2014). It is crucial to take into consideration these different approaches when forming a clear model of neuronal correlates of depressive rumination.

Activity of the DLPFC was found to be inversely correlated with depressive ruminations (Kühn et al., 2012). The role of the DLPFC has been associated with suppressing unwanted thoughts (Kühn et al., 2012) or effective disengagement from the negative material (Vanderhasselt, Kühn, & De Raedt, 2011). The fMRI experiment of Cooney et al. (2010) involved a rumination induction task and two control conditions: concrete and abstract distractions. Each condition included ten statements. For example, “Think about what people notice about your personality” – for rumination; “Think about what contributes to team spirit” – for abstract distraction; and “Think about a row of shampoo bottles on display” – for concrete distraction. Depressed patients had increased activation of the left middle frontal gyrus region (LMFG) compared with healthy controls during rumination versus concrete distraction condition. In rumination versus abstract distraction condition, depressed patients showed different activation patterns of DLPFC subregions than the healthy controls. Healthy subjects exhibited increased activation of the right inferior frontal gyrus (IFG) of the DLPFC, while depressed patients displayed increased activation of the bilateral MFG. Another study, which was conducted only on non-clinical subjects, involved voxel-based morphometry (VBM) and resting state fMRI (Kühn et al., 2012). It used the Ruminative Response Scale (RRS) questionnaire to measure if individuals were prone to depressive ruminations. The RRS scores were found to be negatively correlated with both gray matter volume in the bilateral IFG and resting state activation of this region. Another study conducted using the regional homogeneity (ReHo) method showed that tendency to ruminate is negatively correlated with the functional homogeneity of DLPFC (Wang et al., 2015). Decreased DLPFC activation is in line with most of the neuroimaging studies on brain functional abnormalities in depressive disorder (Fitzgerald, Laird, Maller, & Daskalakis, 2008; Kross, Davidson, Weber, & Ochsner, 2009). De Raedt and Koster (2010) proposed a framework on cognitive vulnerability for depression, which begins with HPA (hypothalamic–pituitary–adrenal) axis hyperactivation, leading to a decrease in PFC activity. This results in attenuated inhibition of subcortical regions, like the amygdala, and a sustained negative affect. The exact ruminative activity pattern in this region is still ambiguous, as a study by Cooney et al. (2010) has revealed hyperactivation of the DLPFC, which would seem to contradict this model. Discrepancies in the obtained results may be related to the functional differences between subregions of the DLPFC. The IFG activation and size were found to be decreased in ruminating individuals (Kühn et al., 2012), while the MFG activation was increased in depressed individuals when ruminating (Cooney et al., 2010).

Activity in the anterior cingulate cortex (ACC) has also been indicated as a depressive rumination correlate. The anterior cingulate is specialized in the regulation of autonomic structures during emotional arousal. It also “inspects” if it is necessary to increase cognitive control and sends this information to the DLPFC (Cohen, Botvinick, & Carter, 2000). The ACC was shown to be more activated in depressed patients during rumination versus both concrete and abstract conditions (Cooney et al., 2010). In the experiment by Kühn et al. (2012), the tendency to ruminate in healthy subjects was negatively correlated with the resting state activation and gray matter volume of ACC as measured by the VBM. Discrepancies of the studies regarding the ACC may be related to the heterogeneity of the ACC subregions. The ventral part of the ACC (vACC) has numerous connections with the amygdala and is sensitive to emotional stimuli, while the dorsal ACC, which controls the activation of the vACC, is more related to cognitive and regulatory processes (Bush, Luu, & Posner, 2000). Further research is required to determine the role of the ACC in depressive ruminations.

Studies regarding the temporal cortex, amygdala, and hippocampus are more consistent. Cooney et al. (2010) found enhanced amygdala, parahippocampal, and temporal gyri (middle temporal gyrus and superior temporal gyrus) activation in depressed patients compared to healthy controls during the ruminative versus abstract condition. Increased or sustained activation of the amygdala during ruminations was repeatedly observed. The fMRI study of passive viewing of negative and neutral images by Ray et al. (2005) revealed a positive correlation between left amygdala activation and the tendency to ruminate. It was also shown that depressed patients can be characterized by sustained amygdala activity after negative emotional processing in comparison to never-depressed controls. This difference was moderately related to the tendency to ruminate (Siegle, Steinhauer, Thase, Stenger, & Carter, 2002). A similar result was found in another fMRI study by Mandell et al. (2014). Trait rumination co-varied with increased amygdala activity. Additionally, after controlling for the amygdala, bilateral hippocampus activation was found to be associated with ruminative tendencies. Increased amygdala activation may be related to heightened emotional reactivity in the face of the negative, self-referential stimuli. The amygdala can also facilitate the retrieval of the emotional memory by modulating the activity in the hippocampus (Disner, Beevers, Haigh, & Beck, 2011). Orbitofrontal cortex (OFC) activation may also be related to emotional reactivity, as it was found that its activation correlates with the subjective estimation of aversive affective state (Garrett & Maddock, 2006) and it has robust connections with the amygdala (Barbas, 2007). While ruminating versus thinking about concrete images, depressed patients were shown to exhibit increased activation of the OFC in comparison to healthy controls (Cooney et al., 2010). Ray et al. (2005) compared brain activities of two non-clinical groups – individuals with a high versus A low tendency to ruminate during the presentation of the IAPS (International Affective Picture System) pictures. Their fMRI study revealed that ruminators were characterized by increased activation of Brodmann area 47, a subregion of the orbitofrontal cortex, when instructed to increase their negative affect and during passive viewing of the negative pictures (Ray et al., 2005). Individuals with high tendency to ruminate may perceive the same stimuli as subjectively more negative than those who do not ruminate.

To conclude the primary objective of this study, we aimed at identifying neuronal correlates of the rumination trait, by examining the postulated emotional control network. We compared the brain activity of people scoring high (RUMINATORS) and low (NONRUMINATORS) on the Ruminative Response Scale Revised (RRS-R) during ruminations induced in a laboratory. Apart from the ruminative condition, we used two control conditions (positive and neutral reflection). The existing literature supports the idea that ruminations may be related to the abnormal functioning of the brain’s emotional regulation network. However, the exact model of the neural basis of depressive ruminations and their detailed functional significance is still unknown. Tendency to ruminate is associated with inability to control intrusive, repetitive thoughts of negative value. Thus, we predicted that the impaired top-down cognitive control in RUMINATORS is a key factor resulting in maladaptive ruminations. We hypothesized that RUMINATORS in comparison to NONRUMINATORS would be characterized by decreased activation of the DLPFC and that information flow from this structure to other regions of the emotional control circuit will be decreased. As a result, attenuated modulatory influences will be associated with increased temporal activation, which is densely interconnected and functionally linked with the limbic structures such as the amygdala and hippocampus. We expect that they will be mostly pronounced in the depressive rumination condition, which may reflect a specific negative bias when retrieving emotional memories or increased emotional reactivity when processing negative self-referential material. We also speculated that RUMINATORS in comparison to NONRUMINATORS will exhibit increased activation of the OFC, especially in the depressive rumination condition. Increased activation in this region may reflect heightened encoding of the affective relevance or arousing properties of a negative stimuli by RUMINATORS (Ray et al., 2005). EEG spatial resolution is insufficient to differentiate between dorsal/ventral ACC, which serve different processes (Cai & Padoa-Schioppa, 2012), and that is why no directional hypothesis related to the ACC was proposed.

In order to test our hypotheses, we applied two separate analytic methods based on the EEG recording. Firstly, in order to identify and trace the activity of functionally-independent brain sources, we applied the Independent Components Analysis (ICA) together with DIPFIT, as a source localization method. Most of the previous rumination research was conducted using the fMRI, so this approach has not been used before. The important advantage of the EEG recording is that it is a more natural environment for the individual than a measurement in the fMRI scanner. The fMRI scanner noise and general physical discomfort related to being confined in a small space may influence the emotional state of the subjects. Secondly, the EEG method provides a unique opportunity to calculate the effective brain connectivity. Hence, the direction and intensity of the information flow within the emotional regulation circuit were also examined.

Two questionnaires were used in our study. The first was the Ruminative Response Scale Revised (RRS-R), used to measure the tendency to ruminate and to divide participants into RUMINATORS and NONRUMINATORS. This scale, contrary to the original Ruminative Response Scale (RRS) includes only items that are not confounded with the depressive content. This approach enabled us to minimize possible influences of general depressive symptoms. The second questionnaire was the Difficulties in Emotional Regulation Scale (DERS), used to measure diversified aspects of the emotional regulation like: acceptance of one’s emotions, awareness and understanding of emotional responses, access to effective emotional regulation strategies and ability to control impulsive behavior and to engage in the goal directed behavior when feeling depressed (Gratz & Roemer, 2004). As this scale is based on multifaceted and comprehensive conceptualization of the emotional regulation, we were able to further determine behavioral specificity of our findings. We did that by testing the relationship of DERS subscales and effective connectivity values when controlling for RRS-R. We expected that our results would be driven specifically by the depressive rumination construct rather than other general cognitive processes involved in the emotional regulation.

## Materials and methods

### Questionnaires

The Ruminative Response Scale (Nolen-Hoeksema & Morrow, 1991) is a questionnaire used to measure the tendency to ruminate. It consists of 22 items that describe individual responses to depressed mood, which can be divided into: self-focused (e.g., I think: “Why do I react this way?”); symptom-focused (I think about how hard it is to concentrate), and consequences/causes focused (I think “I won’t be able to do my job if I don’t snap out of this”). The Ruminative Response Scale Revised (RRS-R) is a modified, 10-item version of the RRS scale (Treynor, Gonzalez, & Nolen-Hoeksema, 2003), which incorporates only those questions which do not overlap with the 13-item BDI (Beck Depression Index). Participants decide, on a 1–4 Likert scale, how often they experience each type of thoughts (1 – almost never; 4 – almost always). Highest possible score is 40.

The Difficulties in Emotional Regulation Scale (Gratz & Roemer, 2004) is a psychometric tool used for complex measurements of emotional regulation disabilities. It includes 36 items assigned to one of six dimensions/subscales (Non-acceptance of Emotional Responses; Difficulties Engaging in Goal-Directed Behavior; Impulse Control Difficulties; Lack of Emotional Awareness; Limited Access to Emotion Regulation Strategies, Lack of Emotional Clarity). For a detailed description of each subscale see Gratz and Roemer (2004). Participants choose answer on a 1–5 Likert scale (1 – almost never; 5 – almost always). Highest possible score is 180.

### Participants

Participants were recruited based on the Ruminative Response Scale Revised (RRS-R) questionnaire measuring the tendency to ruminate unconfounded with general depression symptoms. 243 students from the Jagiellonian University completed the RRS-R. Only 26 of them, scoring low or high on a scale, took part in the main EEG experiment (22 females and four males; *MEAN* RRS-R total = 25.42, *SD* RRS-R = 5.85). In order to enhance possible group differences, we focused on those participants who had more than one standard deviation above the average score (RUMINATORS; high tendency to ruminate; *N* = 11; *MEAN* RRS-R = 34.36; *SD* RRS-R = 2.06) or one standard deviation below the average score (NONRUMINATORS; low tendency to ruminate; *N* = 15, *MEAN* RRS-R = 17.33; *SD* RRS-R = 3.52).

### EEG equipment

Experimental data were collected using a 64-channel EEG BioSemi Active Two acquisition system, sampled with 256Hz frequency. We used the International 10-20 System of Electrode Placement with the adjustment to the nasion and inion. Two additional leads were present on the left and right mastoids (for off-line re-referencing). All electrodes’ impedance was kept in the recommended range during the entire recording.

### Experimental procedure

Experimental procedure was compliant with the directives of the Helsinki Declaration and approved by the ethics committee at the Jagiellonian University’s Institute of Psychology. Subjects signed written consent forms. The EEG measurement took place in an air-conditioned and soundproof room. The experimental task was based on emotional mental imagery. There were three within-subjects conditions: (1) negative (depressive), (2) positive, and (3) neutral. Voice instructions played by a computer were used to induce each type of mental imagery. For example “*Think about a mistake you have recently made”* to evoke the depressive ruminative state, *“Think about an old wooden door”* for the neutral condition, and *“Think about one of the happiest moments in your life”* for the positive one. There were 22 statements all together, and they were mixed in a random order with the exception that the last two statements were always positive. Participants were instructed to close their eyes at the beginning of the procedure and listen to the commands; each one was followed by 30 s of silence intended for a particular imagery task. After the main procedure, subjects were asked to evaluate on a three-point scale of 0–2 (0 – failure; 1 – completed task, but experienced problems with concentration; 2 – success) whether they managed to complete each imagery task. Tasks rated as 0 were not included in the following analysis. Finally, subjects completed the DERS questionnaire.

### Data analysis

#### EEG data: (1) Spectral and source localization analyses of independent EEG components

Preprocessing of the EEG data was performed in the EEGLAB Matlab toolbox (Delorme & Makeig, 2004). The signal was referenced off-line to the linked mastoids and filtered (1-46 Hz, zero-phase). Single data channels with severe technical failures (excessive noise, prolonged loss of skin connection) were then rejected based on visual inspection. No participant had more than two data channels rejected. Each 30-s fragment associated with the imagery task was divided into 2-s epochs with a 0.5-s overlap. Epochs which involved apparent artifacts (muscles, technical problems, eye movements) were rejected on the basis of the semi-automatic procedure. First, an automatic algorithm was applied, which involved both threshold level (below −80 μV and over +150 μV) and abnormal spectra (excessive power in beta/gamma frequencies with threshold set to 30 dB above the electrode average in the 25–45 Hz range on scalp electrodes). Then, the final visual inspection was performed to confirm the rejection. As a result, the average number of epochs taken for analysis in a single subject was 331 and the minimum number was 158.

A blind source separation algorithm, Independent Component Analysis (ICA), was carried out on the remaining EEG signal in order to identify independent brain sources contributing to the scalp electrical signal. The independent components (IC) originating in oculomotor activity and eye-blinks were rejected from further analysis on the basis of typical artifactual parameters (spatial distribution, time characteristics relative to stimuli onset and spectral power; Jung et al., 2000).

The equivalent source dipoles of the identified independent components were localized using the DIPFIT2 method based on a standardized boundary element head model (BEM). This procedure uses an averaged, re-referenced EEG signal and consists of an initial coarse model grid search, followed by non-linear fine fitting, with an option to search for either single or bilateral dipoles (Kybic, Clerc, Faugeras, & Keriven, 2006; Niedermeyer, 1996). Those dipole localizations that best fit the signal distribution on the scalp surface were chosen. Only those ICs whose residual variance (RV) of dipole location was less than 15 % were taken into consideration during the following analysis (Wyczesany, Grzybowski, & Kaiser, 2015). For each remaining independent component, spectral analysis was performed. Absolute spectral power density (expressed in μV^2^/Hz) was computed using FFT algorithm, with 10 % Hanning window applied to each 2-s epoch and then averaged. In order to identify functionally corresponding ICs in all the subjects, the K-means clustering method was performed. The clusters were computed on the basis of spatial location (weight: 2/3) and power spectrum similarity (weight: 1/3) using least-squares Euclidian distances (MATLAB k-means method) and reduction of the resulting vector to 10 dimensions using principal component analysis (PCA). The initial number of searched clusters was set for k = 20 and the threshold level for outliers remained at 2.5 SD of the estimated distance. The clustering procedure was iteratively repeated with k decreasing by one in each run, until they were judged to remain functionally and anatomically distinct with regard to functional plausibility determined by correspondence with anatomical structures (Jung et al., 2007; Wyczesany et al., 2015).

The obtained power spectra density values were aggregated into the following bands: alpha (8–15 Hz); beta1 (13–15 Hz); beta2 (16–24 Hz); beta3 (25–30 Hz). All spectral values were averaged within the specific condition (negative/depressive, positive, and neutral). As beta oscillations are the ones that are most functionally heterogeneous (Rangaswamy et al., 2002), the frequency range was divided into three smaller subunits. The resulting data (alpha, beta1, beta2, and beta3 power spectra density values) were compared between groups (RUMINATORS/NONRUMINATORS) with a within-subject valence conditions factor (neutral, positive, depressive; mixed-design ANOVA).

#### EEG data: (2) Effective connectivity

The Directed Transfer Function (DTF; Korzeniewska, Mańczak, Kamiński, Blinowska, & Kasicki, 2003) is a multivariate autoregressive modeling (MVAR) method for assessing effective connectivity, using Granger causality principles. It provides a multivariate, causal estimation of the information flow rate and direction while controlling the family-wise alpha level. According to the MVAR model, each data sample in k channels at a time t can be expressed as a weighted sum of p previous samples with a random component added:

$$ \mathbf{X}(t)={\displaystyle \sum_{j=1}^p\mathbf{A}(j)\mathbf{X}\left(t-j\right)+\mathbf{E}(t)} $$

where **X**(t) is the data values vector and **E**(t) is the random component values vector at the time t. The **A**(j) is the MVAR model coefficients matrix and p is the model order, which is equal to the number of past samples used to model the signal. The MVAR model can be transformed into the frequency domain:

$$ \begin{array}{c}\hfill X(f)={A}^{-1}(f)E(f)=H(f)E(f)\hfill \\ {}\hfill H(f)={\left({\displaystyle \sum_{m=0}^p}A(m) exp\left(-2\pi imf\varDelta t\right)\right)}^{-1}\hfill \end{array} $$

where **X**(f), **A**(f) and **E**(f) are the Fourier transform of **X**(t), **A**(j) and **E**(t) matrices, respectively, and the matrix **H**(f) = **A** − 1(f) is known as the transfer matrix. The DTF function can be expressed as:

$$ {\gamma}_{ij}^2(f)=\left|{H}_{ij}(f)\right|2 $$

where γ_ij_(f) describes the causal influence of channel j on channel i at frequency f. More details on the method can be found in Kaminski & Blinowska (1991) and Ligeza, Wyczesany, Tymorek, and Kamiński (2015).

DTF calculations were made using Multar software (Department of Biomedical Physics, University of Warsaw). The method can only be used on original electrodes’ signal (and not on linear combinations of signals like independent components). However, as it is based on autoregressive modeling, it is insensitive to the volume conduction phenomenon, which provides increased spatial resolution (Kaminski & Blinowska, 2014). Therefore, instead of using independent sources, we examined effective connectivity between electrodes over the regions of interest mentioned in the hypotheses. Based on EEG montage brain atlases (Kaiser 2007; Okamoto et al. 2004), electrodes corresponding to our regions of interest were selected as follows: left DLPFC (LDLPFC: F3); right DLPFC (RDLPFC: F4), ACC (Fz); left temporal area (LTmp: T7, TP7); and right temporal area (RTmp: T8, TP8). The DTF values were calculated for the whole beta band, as this frequency window covers an important part of middle- and long-range cortical communication (Kuś, Blinowska, Kamiński, & Basińska-Starzycka, 2008; Wyczesany et al., 2014a). Group and valence differences in effective connectivity were examined for the following ROIs in both directions separately: LDLPFC↔LTmp; RDLPFCR↔RTmp; LDLPFC↔RTmp; RDLPFC↔LTmp; LDLPFC↔ACC; RDLPFC↔ACC; (repeated-measures MANOVA).

#### Questionnaires

The total scores of the RRS-R questionnaires were correlated with the DERS questionnaire subscales using the Spearman correlation coefficient (as the scale distribution shifted towards bimodal distribution due to participant enrolment procedure to the final group). Spearman partial correlations of DERS subscales and effective connectivity values were also tested when controlling for RRS-R.

## Results

### EEG data: (1) Spectral and source localization analyses of independent EEG components

**Fig. 1:**
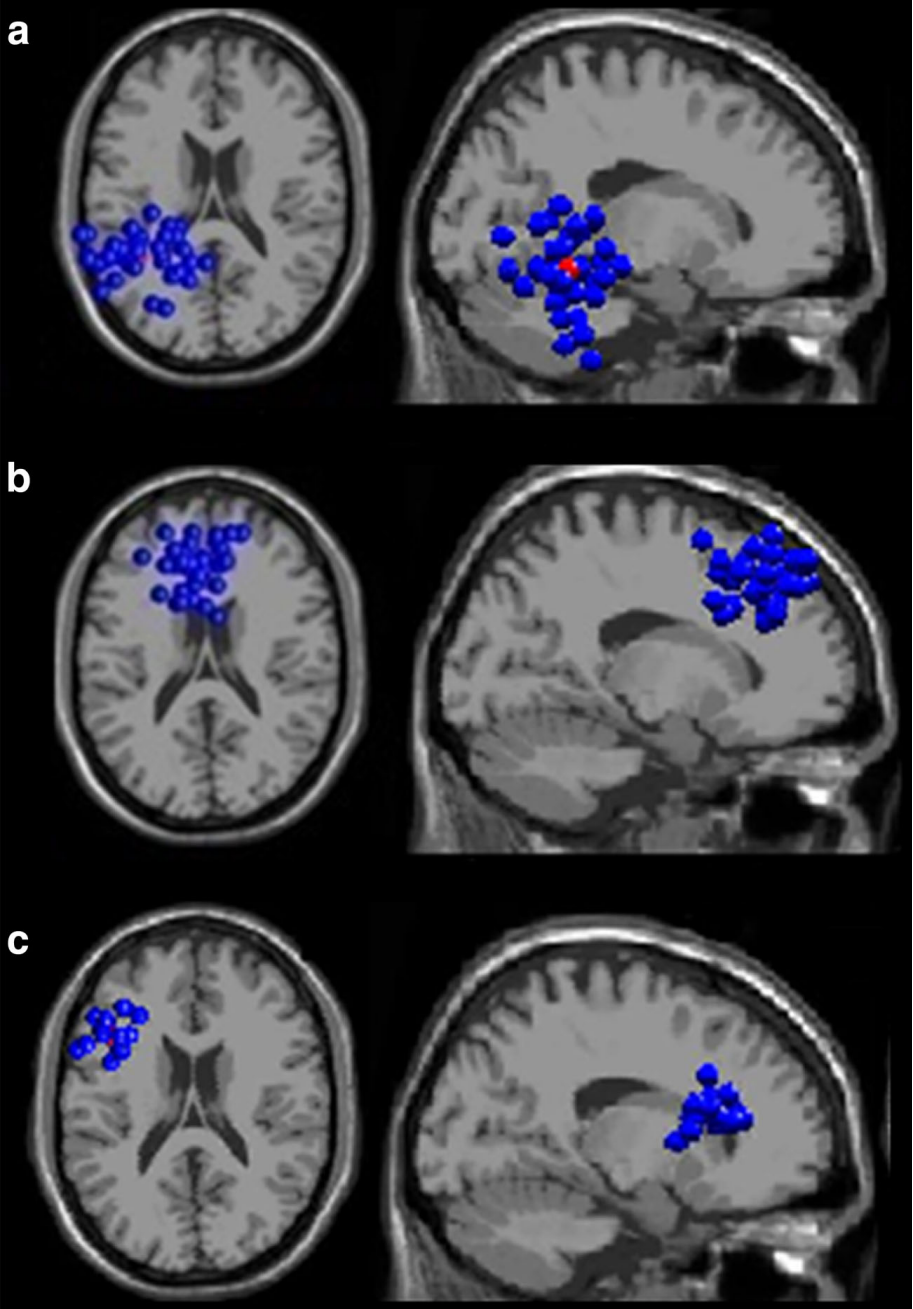
Localization of the three clusters of equivalent source dipoles (blue points denote dipoles of constitutive independent components as localized in each subject, while the red one denotes the cluster centroid); (**a**) the left temporal cortex (LTmp) cluster, top and saggital view; (**b**) the anterior cingulate cortex (ACC) cluster, top and saggital view; (**c**) the left dorsolateral prefrontal cortex (LDLPFC) cluster, top and saggital view. Clusters were modelled with the use of the MNI standard brain template

As a result of the independent component clustering, 16 clusters of independent sources were identified. Their exact localizations are shown in Table 1. Group effects of spectral power were revealed in three clusters of sources, localized in LTmp, ACC and LDLPFC (localizations of each cluster’s sources are presented in Fig. 1). An additional group*valence interactive effect was found in the LTmp region.

**Table 1:** Parameters of independent components clusters with their localization (referring to cluster centroid) in Talairach coordinates

Cluster no.				Area	Brodmann area	mean RV [%]
1	−39	−45	−11	Left Fusiform Gyrus (lTmp) left posterior temporal	37	6,49
2	−5	−96	1	Left Cuneus	17	9,25
3	45	5	−37	Right Middle Temporal Gyrus	38	5,46
4	−5	35	39	Cingulate Gyrus (ACC)	8	5,83
5	−53	−35	16	Left Insula	13	6,50
6	−17	10	−13	Left Inferior Frontal Gyrus	47	9,94
7	−22	−5	10	Left Putamen		9,01
8	12	−19	6	Right Thalamus		9,99
9	−60	−12	−11	Left Middle Temporal Gyrus	21	7,31
10	16	16	16	Right Caudate		9,00
11	1	−42	43	Left Precuneus	7	4,87
12	−27	−10	40	Left Middle Frontal Gyrus	6	7,63
13	−42	20	14	Left Inferior Frontal Gyrus (lDLPFC)	45	7,25
14	−53	24	−3	Left Inferior Frontal Gyrus (OFC)	47	6,20
15	−16	55	−3	Left Superior Frontal Gyrus	10	7,14
16	−56	−5	11	Left Precentral Gyrus	43	7,05

*RV* random variation

**Fig. 2:**
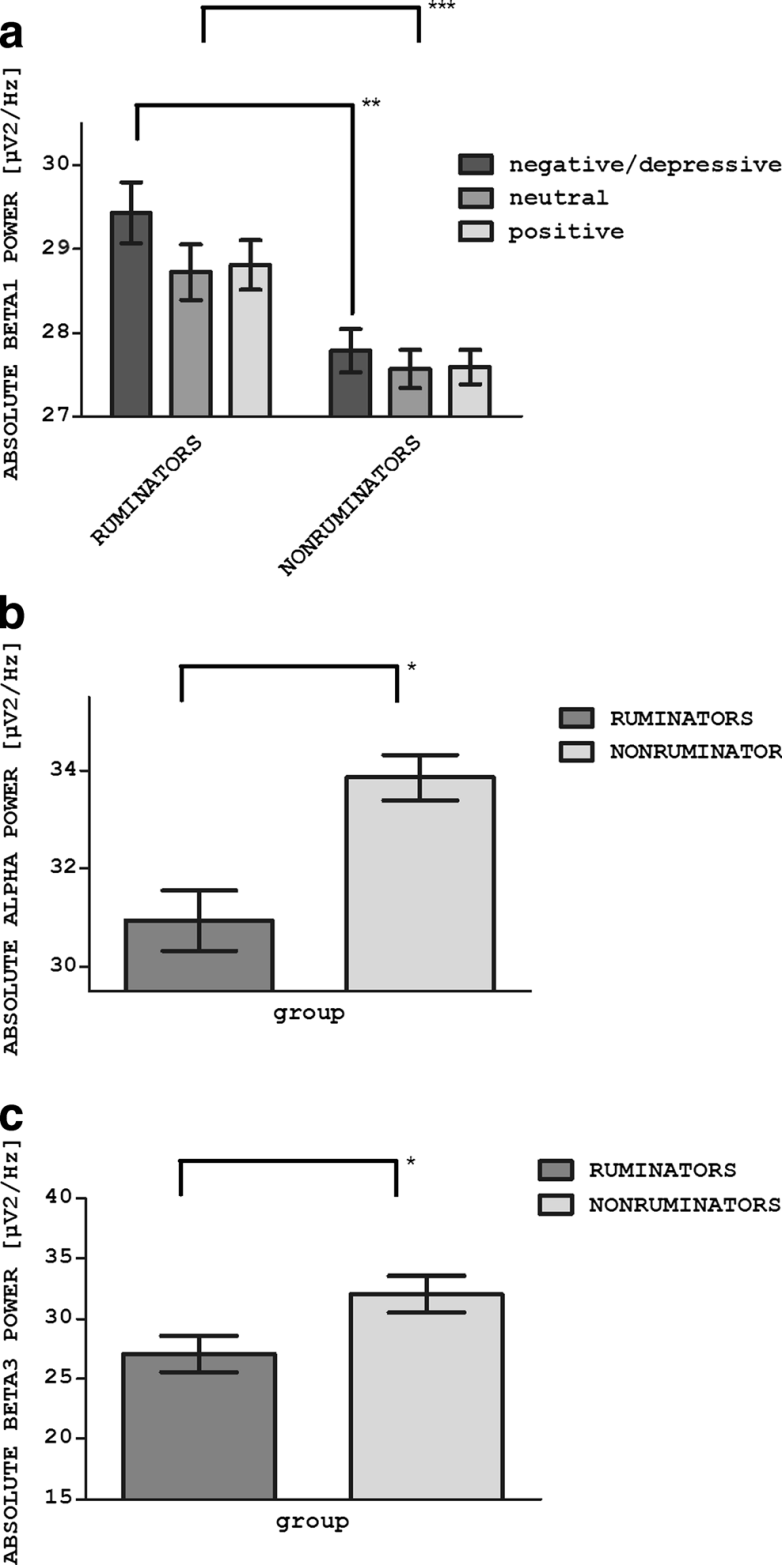
Group effects for RUMINATORS / NONRUMINATORS comparisons. The bars represent spectral power values for the considered IC clusters. Error bars represent ±1 SE. **a** The group effect for the beta1 power (***) and the group*valence interaction effect (**) for the beta1 power in the left temporal cortex and the (LTmp) cluster. **b** The group effect for the alpha power in the anterior cingulate cortex (ACC) cluster (*). **c** The group effect for the beta3 power in the left dorsolateral prefrontal cortex (LDLPFC) cluster (*). *** *p* ≤ 0.001; ** *p* ≤ 0.01; * *p* ≤ 0.05

In the left temporal cortex (LTmp) cluster, for the beta1 power, a significant main group effect (RUMINATORS / NONRUMINATORS) was found. RUMINATORS, in comparison to NONRUMINATORS, were characterized by higher overall beta1 power (*F*(1,54) = 11.79; *p* = .001). Moreover, a group*valence interactive effects were observed: *F*(1.725, 93.143) = 5.41*; p* = .008*;* Greenhouse-Geisser correction applied; Fig. 2). Simple effect analysis revealed a significantly higher beta1 power in the RUMINATORS (than in NONRUMINATORS) for depressive rumination compared to the positive condition (*p* = .026) and for depressive rumination compared to the neutral condition (*p* = .004).

In the anterior cingulate cortex (ACC) cluster, a main group effect was found in the alpha frequency range. RUMINATORS, in comparison to NONRUMINATORS, were characterized by a lower spectral power value in the alpha band in all experimental conditions (*F*(1,26) = 14.28; *p* = .001; Fig. 2).

In the left dorsolateral prefrontal cortex (LDLPFC) cluster, statistical analysis revealed a main group effect in beta3 power. RUMINATORS, in comparison to NON-RUMINATORS, were characterized by lower beta3 power in all experimental conditions (F(1,12) = 5.44; p = .038; Fig. 2).

Contrary to our hypotheses, no group differences (RUMINATORS / NONRUMINATORS) were found in the OFC area.

Apart from our clusters of interest (those related to the hypotheses), three other clusters revealed by the ICA, DIPFIT, and cluster analyses were characterized by the particularly low residual variance. These were located in the right middle temporal gyrus, left precuneus, and left insula. They were additionally checked for any group differences, but this check gave negative results. No differences between RUMINATORS and NONRUMINATORS were found (for detailed statistics please see the Supplemental Materials). Some valence effects were only identified, differentiating between neutral and emotional conditions or only between neutral and ruminative conditions. In the left precuneus cluster, a valence effect was found in beta1 power (*F*(2,54) = 6.23; *p* = .004). In the left insula cluster, valence effects were present in the alpha (*F*(1.646, 62.535) = 4.67; *p* = .018), beta1 (*F*(2, 76) = 11.28; *p* < .001) and beta2 (*F*(2, 76) = 5.08; *p* = .009) power.

### EEG data: (2) Effective connectivity

DTF analysis revealed decreased beta information flow from the RDLPFC to the LTmp in RUMINATORS compared to NONRUMINATORS (*F*(1,23) = 4.73; *p* = .040). LDLPFC to the LTmp beta information flow differences between RUMINATORS and NONRUMINATORS were not significant (*F*(1,21) = 1.19; *p* = .288), but as the channel*group interactive effect was statistically significant (*F*(1,42) = 4.32; *p* = .050), we have checked the simple effects for each temporal channel (T7 and TP7). These analyses revealed that the group effect was loaded by the LDLPFC (F3) to the posterior LTmp (TP7) [F3 → TP7] flow (*F*(2,21) = 6.44; *p* = .019), while the LDLPFC (F3) to the anterior LTmp (T7) flow did not differ according to the group (*F*(2,21) = 0.23; *p* = .636). This was the only direction with the significant channel*group interactive effect (statistics for other directions can be found in Supplemental Materials).

**Fig. 3:**
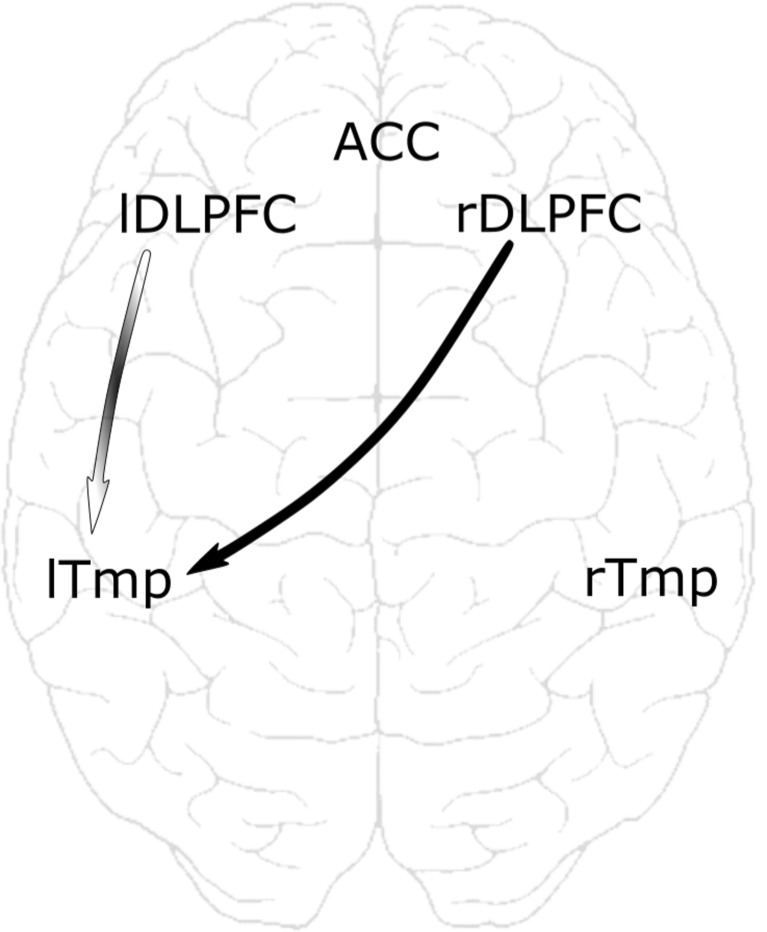
The flows between brain regions are represented by the arrows. Group effects which were significant in RUMINATORS / NONRUMINATORS comparisons are represented by the black arrows (*p* < .05). The arrow with the gradient filling represents the flow where electrode*group interaction and simple effect for F3 → TP7 electrodes (LDLPFC to posterior LTmp) was significant. LDLPFC = left dorsolateral prefrontal cortex; RDLPFC = right dorsolateral prefrontal cortex; LTmp = left temporal cortex; RTmp = right temporal cortex; ACC – anterior cingulate cortex

Interestingly, we did not find group differences in the opposite directions – from temporal regions to dorsolateral prefrontal cortex (LTmp → RDLPFC and LTmp → LDLPFC). No significant between-group differences in beta information flow were found in the following directions either: RDLPFC → RTmp; LDLPFC → ACC; RDLPFC → ACC; ACC → LDLPFC; ACC → RDLPFC; RDLPFC;; RTmp → RDLPFC; RTmp → LDLPFC. All connectivity values and significance levels are given in Table 2 and a topographic view of the flows that differentiate between RUMINATORS / NONRUMINATORS is shown in Fig. 3.

**Table 2:** The connectivity values between the areas of interest

Direction	DTF RUM	DTF NONRUM	*p* value
RDLPFC → RTmp	22,859	27,708	*p* = .932
RDLPFC → LTmp	5,420	10,117	*p* = .040*
LDLPFC → LTmp	10,559	16,593	*p* = .288 (F3 → TP7;p = .019)*
LDLPFC → RTmp	6,480	10,115	*p* = .146
RDLPFC → ACC	29,963	25,722	*p* = .716
LDPFC → ACC	43,389	37,656	*p* = .639
RTmp → RDLPFC	22,859	27,708	*p* = .598
LTmp → RDLPFC	23,000	15,816	*p* = .408
LTmp → LDLPFC	24,803	23,524	*p* = .903
RTmp → LDLPFC	30,760	25,419	*p* = .623
ACC → RDLPFC	28,413	31,579	*p* = .801
ACC → LDLPFC	12,181	20,699	*p* = .116

*RUM* RUMINATORS, *NONRUM* NONRUMINATORS, *DTF* Directed Transfer Function value

* *p* ≤ 0.05

### Questionnaires

Since the Shapiro-Wilk tests revealed that normality distribution assumption was not achieved, Spearman correlations were performed. Statistically significant positive or negative correlations were found between RRS-R and all DERS subscales. Detailed statistics are given in Table 3. Additionally, partial correlations of the effective connectivity measures and DERS subscales were examined, while controlling for the RRS-R score. Most of the correlations were not significant, with the exception of the correlation of the Difficulties Engaging in Goal-Directed Behavior DERS subscale and the effective connectivity measures from the RDLPFC to the LTmp (averaged beta information flow from F4 to T7 and from F4 to TP7 electrodes). The effective connectivity from the LDLPFC to the LTmp did not correlate with any of the DERS subscales. Detailed statistics are reported in Table 4.

**Table 3:** Spearman correlations of DERS questionnaire subscales scores and the RRS-R questionnaire scores

	RRS-R
NONACCEPT	r_s_(24) = 0.52; p = .004 **
GOALS	*r*_*s*_(24) = 0.49; *p* = .006 **
IMPULSE	*r*_*s*_(24) = 0.61; *p* = .001 ***
AWARENESS	*r*_*s*_(24) = -0.57; *p* = .001 ***
STRATEGIES	*r*_*s*_(24) = 0.66; *p* < .001 ***
CLARITY	*r*_*s*_(24) = .37; *p* = .032 *

* *p* ≤ 0.05; **p ≤ 0.01;***p ≤ 0.001

**Table 4:** Partial correlations of DERS questionnaire (total and the subscales) and effective connectivity measures with the RRS-R a controlling variable

Partial correlations (controlled for RRS-R variable)	LDLPFC → LTmp (F3 → TP7)	RDLPFC → LTmp
DERS	r_s_ (20) = −1.18; *p* = .416	r_s_ (20) = − .24; *p* = .284
NONACCEPT	r_s_ (20) = −.28; *p* = .212	r_s_ (20) = .04; *p* = .854
GOALS	r_s_ (20) = −.09; *p* = .682	r_s_ (20) = −.43; *p* = .048*
IMPULSE	r_s_ (20) = .01; *p* = .973	r_s_ (20) = −.34; *p* = .072
AWARENESS	r_s_ (20) = .21; *p* = .360	r_s_ (20) = .2; *p* = .660
STRATEGIES	r_s_ (20) = . − .30; *p* = .175	r_s_ (20) = −.30; *p* = .176
CLARITY	r_s_ (20) = .09; *p* = .708	r_s_ (20) = −.14; *p* = .533

*GOALS* Difficulties Engaging in Goal-Directed Behavior, *IMPULSE* Impulse Control Difficulties, *AWARENESS* Lack of Emotional Awareness, *STRATEGIES* Limited Access to Emotion Regulation Strategies, *CLARITY*- Lack of Emotional Clarity

* *p* ≤ 0.05

## Discussion

Our research was aimed at identifying neuronal correlates of depressive rumination in the context of the brain’s emotional control network. Our experimental design enabled us to study the neuronal correlates of both the trait of rumination as well as the ruminative state induced in the laboratory. We have divided our healthy participants into two groups on the basis of the Ruminative Response Scale Revised (RRS-R). As there is an ongoing debate that a strong relationship between depression and rumination results from the overlap of the items between RRS and BDI, Teynor et al. (2003) constructed a revised version of the original RRS, reduced by the 12 items that overlapped with the 13-item BDI. Thanks to using this revised version of the RRS, we were able to prove that our results are related to the depressive rumination construct rather than to the general depressive symptoms. To our knowledge, the EEG independent component method with DIPFIT source localization has been used to study the neurobiological basis of depressive rumination for the first time. Compared to the fMRI study, EEG allows for much more natural experimental settings than the uncomfortable scanner conditions. What is even more important, EEG is also suitable to directly assess effective connectivity, so we were able to verify the direction of information flow between the postulated nodes of the control network and infer about the causality of these influences. This would uniquely contribute to our knowledge of affective modulation, and go beyond the typical fMRI correlational data. Using such novel methodology, we were able to confirm that left dorsolateral hypoactivation and decreased information flow from this structure to temporal regions is a crucial neuronal correlate of a tendency to ruminate.

### EEG spectral power and effective connectivity

RUMINATORS, in comparison to NONRUMINATORS, were characterized by increased activation in the left temporal cortex region. Additionally, the activation of this region was higher for the depressive rumination condition than for the positive and neutral conditions in RUMINATORS compared to NONRUMINATORS, as evidenced by a higher beta1 power. This interactive effect indicates a negative content hyperreactivity, which characterizes ruminating individuals. The left temporal structures are considered to be a part of the emotional memory system (LaBar & Cabeza, 2006). Increased activity in this region in the depressive rumination condition may indicate a more effective retrieval of negatively-valenced memories or higher emotional value attributed to the recalled memories. As the temporal cortex is densely interconnected and functionally linked with the amygdala and hippocampus, the increased activation of this region might have been influenced by those limbic subcortical structures (Wilson et al., 1991). A previous fMRI experiment revealed the rumination-related increase in the amygdala, hippocampus, and parahippocampal areas (Cooney et al., 2010; Mandell et al., 2014; Ray et al., 2005; Siegle et al., 2002).

Increased alpha power in the ACC was also found in RUMINATORS compared to NONRUMINATORS. As the alpha power is an inverse indicator of activation, collected data reveal increased activity in the ACC area in the RUMINATORS group when compared to the NON-RUMINATORS. ACC integrates different aspects of the emotional experience involving autonomic arousal. Increased activation in this region may imply that RUMINATORS experience a greater degree of emotions. Unfortunately, the EEG spatial resolution is not precise enough to examine the activity of functionally distinct subregions of the ACC. The dorsal part of the ACC is involved in regulatory processes, while the ventral part activates automatically when emotional stimuli are presented (Bush et al., 2000). Because of this heterogeneity, our results are difficult to interpret. However, Cooney et al. (2010) found in their fMRI study that depressed patients are characterized by increased activation of the ACC while ruminating, compared to healthy controls. They suggest that this result may be related to the increased self-focus in highly ruminating individuals, as it was previously found that the activity of the rostral part of ACC was increased when healthy subjects were attending to subjective feeling states (Lane, Fink, Chau, & Dolan, 1997). No effect in information flow from the DLPFC to the ACC was found in our study. The lack of significant differences between groups may be due to the fact that the ACC is a relatively deep structure, which is difficult to access with surface EEG recording. It is more accurate to quantify its activity based on the source reconstruction methods than on the direct channel measures used by the DTF method.

The hypothesis regarding group differences in the OFC activity was not confirmed. Possibly, this null result may indicate that the OFC is less related to emotional control, which apparently differs in both groups. However, depressed patients in the Cooney et al. (2010) study were found to exhibit increased activation in OFC regions in the ruminative versus concrete condition in comparison to the control. Therefore, it is also plausible that the effect that is not observed in a non-clinical population in this study will be more pronounced in the clinical group. No group effects were identified in three clusters (right middle temporal gyrus, left precuneus, and left insula clusters), which did not relate to our hypotheses but had low residual variance (below 6.5). Valence effects that were identified in left precuneus and left insula clusters (neutral vs. emotional or neutral vs. ruminative) may indicate that these clusters were only sensitive to the affective aspects of the processed material.

Finally, RUMINATORS were found to be characterized by decreased activation of the left DLPFC when compared to NON-RUMINATORS. Interestingly, no group*valence interactive effect was found, as RUMINATORS showed lower activation in all three conditions. Decreased activation in the group with a high tendency to ruminate was found in the same subregion of the DLPFC (inferior frontal gyrus) as in the Kühn et al. study. The IFG was previously shown to activate when inhibiting unwanted behaviors (Aron, Fletcher, Bullmore, Sahakian, & Robbins, 2003) and during reappraisal of the negative affect (Ochsner et al., 2002). This implies that activation in this region is important for emotional control. The obtained result suggests tonic, relatively stable group characteristics in left DLPFC activations related to the tendency to ruminate. At the level of the disorder’s symptom, prefrontal hypoactivation may be associated with the inability to suppress perseverative tendencies in depressed individuals. This can result in repetitive, negative ruminations. The analysis of the strength of effective connectivity between the DLPFC and the temporal cortices brought more support to our findings. Decreased information flow from the bilateral DLPFC to the temporal cortex was found between RUMINATORS and NONRUMINATORS. This results reinforce the claim about decreased modulatory influence from the DLPFC area in RUMINATORS. The top-down influence of the prefrontal cortex might not be sufficient to modulate temporal cortex activation effectively. As a result of the decreased information flow from the DLPFC to the left temporal cortex, RUMINATORS are not able to down-regulate their negative affect and are more reactive in the face of the negative emotional stimuli. No group differences in information flow from the DLPFC to the ACC were found in our experiment. This may imply that the emotional regulation impairments of RUMINATORS are much more related to the disrupted communication between the DLPFC and temporal cortices, more distant regions involved in emotional control.

### Questionnaires

A high positive correlation between DERS and RRS-R questionnaires indicates a positive relationship between the tendency to ruminate and impaired emotional regulation. A closer look at the correspondence between RRS-R and DERS subscales suggests that RUMINATORS have difficulties with impulse control and with engaging in goal-directed behavior when being depressed. As the main coping strategy of ruminators is to dwell on the negative thoughts in response to the depressed mood, they may not have enough working memory resources to perform different actions effectively. The tendency to ruminate is also associated with lack of emotional clarity, non-acceptance of emotional responses to distress and limited access to emotional regulation strategies. Ruminators neither understand nor accept their emotional states and they use ineffective, maladaptive strategies to regulate their mood. Interestingly however, a negative relationship between Lack of Emotional Awareness DERS subscale and RRS-R can suggest that RUMINATORS are very attentive to their negative feelings. As Bardeen, Fergus, and Orcutt (2012) propose, the Lack of Emotional Awareness DERS subscale may not represent the same emotional regulation mechanism as other DERS subscales do.

We have found a negative relationship between the information flow from the right dorsolateral prefrontal cortex to the left temporal cortex, and difficulties engaging in the goal directed behavior which were not explained by tendency to ruminate. Indeed, the right dorsolateral prefrontal cortex was previously shown to be involved in goal-directed behaviours (Morris, Dezfouli, Griffiths, & Balleine, 2014). Importantly, the effective connectivity between the left dorsolateral prefrontal cortex and the left temporal cortex did not correlate with any of the DERS subscales when controlled for the RRS-R scores. This suggests that circuit localized in the left hemisphere has a specificity for depressive ruminations. This is supported by our spectral power analyses results; differences between RUMINATORS and NONRUMINATORS were left-lateralized and found in the left dorsolateral prefrontal cortex and left temporal cortex.

### Conclusion

We were able to confirm the role of the emotional control circuit in rumination phenomena. Significant differences were found in the left temporal cortex, anterior cingulate cortex, and left dorsolateral prefrontal cortex – structures that are part of the emotional control brain circuit. An interactive effect found in the left temporal cortex may indicate that RUMINATORS attribute higher emotional value to negative memories and are generally more emotionally reactive in the face of negatively valenced, self-referential stimuli. In the ACC region, increased activation was observed in the RUMINATORS group. It can be linked to elevated autonomic arousal or intensified self-focus. Finally, RUMINATORS were characterized by decreased activation of the LDLPFC. This might be a manifestation of the attenuated top-down modulatory influences associated with impaired cognitive emotional control. The DTF analysis confirmed this interpretation by revealing decreased information flow from the bilateral DLPFC to the left temporal cortices in RUMINATORS, compared to NON-RUMINATORS. No differences in information flow from the DLPFC to the ACC were found. Thus, emotional regulation difficulties observed in ruminating individuals might be much more related to the disrupted communication between dorsolateral cortex regions and temporal cortices. Questionnaire results confirm the existence of the relationship between emotional regulation deficits and the tendency to ruminate. Finally, we have also shown that beta information flow from the left dorsolateral prefrontal cortex to the left temporal cortex has a specificity for the depressive rumination. We argue that the dysfunction of the top-down emotional control performed by the left DLPFC on the left temporal cortices is crucial in the context of depressive rumination.

### Relevance of this study

Our experiment was an attempt to find neuronal correlates of the tendency to ruminate. Thanks to our approach, we can conclude that there is a crucial neuronal correlate of the depressive rumination. As we predicted, hyperactivation of the left temporal cortex, hypoactivation of the DLPFC and its ineffective modulatory actions on temporal areas are the main neuronal basis for the tendency to ruminate. Further investigation is required to describe the exact characteristics of the ruminators’ brain activity patterns. As ruminative tendency is a predictor for developing depressive disorder, identification of these markers may be used as an objective method to measure the risk for this disease. Successive brain measurements during ongoing psychological therapy could also serve as a control of the treatment’s effectiveness. Our study emphasizes the relationship between the tendency to ruminate and emotional control abilities. Applying cognitive control training may result in a decreased frequency of ruminations and protect patients from developing depressive disorder (Cohen, Mor, & Henik, 2014). Mindfulness-based cognitive therapy was also shown to be a successful intervention in depressive disorder prevention and in treatment-resistant depressed patients (Kenny & Williams, 2007). During mindfulness meditation, patients are taught to simply observe their thoughts without any emotional judgment. Acceptance and less attachment to one’s thoughts lead to increased control over automatically appearing ruminative thoughts’ patterns (Eisendrath, Chartier, & McLane, 2011). Another effective method for dealing with excessive rumination was studied by Bratman et al. (2015) on healthy participants. Their experiment revealed that a 90-min walk in the nature can reduce rumination as measured by the Reflection Rumination Questionnaire.

### Limitations and Directions for Future Research

The general limitation of the EEG method is its relatively low spatial resolution. Thus, it is important to note that EEG signal sources are only approximately localized. Nevertheless, it was shown that low noise active electrodes can limit the localization shift 1–1.5 cm when using 64 derivations, as in our case (Lelic, Gratkowski, Valeriani, Arendt-Nielsen, & Drewes, 2009). The majority of our participants were females, which could have influenced our results. Nevertheless, according to the meta-analysis by Johnson and Whisman (2013), gender differences in rumination are relatively weak. Another limitation of our research was lack of measurement of depressive symptoms using the BDI (Beck Depression Inventory) questionnaire. Notwithstanding, we have used the revised version of the RRS scale which is not confounded with the depressive content (Lee & Kim, 2014; Treynor et al., 2003) and this enabled us to verify the neuronal correlates of the rumination as a self-standing construct.

In the future, experiments examining the neuronal correlates of depressive ruminations in the clinical population are planned. An opportunity to compare neuronal correlates of depressed ruminators and highly ruminating individuals without the depressive disorder diagnosis would provide an insight into the mechanism of depressive disorder development. It is highly probable that the effects observed in this study would be more pronounced in the clinical population.
